# Plasma-Based Bioinks for Extrusion Bioprinting of Advanced Dressings

**DOI:** 10.3390/biomedicines9081023

**Published:** 2021-08-16

**Authors:** Cristina Del Amo, Arantza Perez-Valle, Miguel Perez-Garrastachu, Ines Jauregui, Noelia Andollo, Jon Arluzea, Pedro Guerrero, Koro de la Caba, Isabel Andia

**Affiliations:** 1 Regenerative Therapies, Bioprinting Laboratory, Biocruces Bizkaia Health Research Institute, Cruces University Hospital, 48903 Barakaldo, Spain; cristina.delamomateos@osakidetza.eus (C.D.A.); arantzaperez6@gmail.com (A.P.-V.); INES.JAUREGUIMONASTERIO@osakidetza.eus (I.J.); 2 Department of Cell Biology and Histology, School of Medicine and Nursing, University of the Basque Country, UPV/EHU, 48940 Leioa, Spain; Mperez282@gmail.com (M.P.-G.); noelia.andollo@ehu.eus (N.A.); jon.arluzea@ehu.eus (J.A.); 3 BEGIKER, BioCruces Bizkaia Health Research Institute, 48903 Barakaldo, Spain; 4 BIOMAT Research Group, Escuela de Ingeniería de Gipuzkoa Donostia-San Sebastián, University of the Basque Country (UPV/EHU), 20018 Donostia-San Sebastian, Spain; pedromanuel.guerrero@ehu.es (P.G.); koro.delacaba@ehu.eus (K.d.l.C.); 5 BCMaterials, Basque Center for Materials, Applications and Nanostructures, UPV/EHU Science Park, 48940 Leioa, Spain

**Keywords:** cytokines, bioprinting, growth factors, platelet-rich plasma, wound healing

## Abstract

Extrusion bioprinting based on the development of novel bioinks offers the possibility of manufacturing clinically useful tools for wound management. In this study, we show the rheological properties and printability outcomes of two advanced dressings based on platelet-rich plasma (PRP) and platelet-poor plasma (PPP) blended with alginate and loaded with dermal fibroblasts. Measurements taken at 1 h, 4 days, and 18 days showed that both the PRP- and PPP-based dressings retain plasma and platelet proteins, which led to the upregulation of angiogenic and immunomodulatory proteins by embedded fibroblasts (e.g., an up to 69-fold increase in vascular endothelial growth factor (VEGF), an up to 188-fold increase in monocyte chemotactic protein 1 (MCP-1), and an up to 456-fold increase in hepatocyte growth factor (HGF) 18 days after printing). Conditioned media harvested from both PRP and PPP constructs stimulated the proliferation of human umbilical vein endothelial cells (HUVECs), whereas only those from PRP dressings stimulated HUVEC migration, which correlated with the VEGF/MCP-1 and VEGF/HGF ratios. Similarly, the advanced dressings increased the level of interleukin-8 and led to a four-fold change in the level of extracellular matrix protein 1. These findings suggest that careful selection of plasma formulations to fabricate wound dressings can enable regulation of the molecular composition of the microenvironment, as well as paracrine interactions, thereby improving the clinical potential of dressings and providing the possibility to tailor each composition to specific wound types and healing stages.

## 1. Introduction

The growing prevalence of chronic wounds is threatening health-system sustainability worldwide, as they represent a “silent epidemic”, with aging and diabetes as major drivers [1]. A 2020 market research report valued the market at $6.6 billion in 2020 and predicts that it will reach $16.36 billion by 2027 [2]. Research on the adoption of novel manufacturing technologies for advanced active-dressing developments can partly address wound-related challenges.

Extrusion bioprinting (also referred to as bioplotting) is an automated biofabrication technology that accommodates cells within hydrogels and involves the extrusion of so-called bioinks by the application of pressure, either pneumatic or piston-driven [3]. Hydrogels are hydrophilic networks of synthetic or natural polymers. For example, collagen, gelatin, alginate, chitosan, hyaluronan, fibrin, and a decellularized extracellular matrix are natural polymers that are typically used as components of bioinks. Formulations of hydrogels or their precursors along with cells and signaling molecules can be used to create preset 3D geometric structures tailored to host tissue for healing and reconstructive medicine purposes [4].

However, technical challenges to the development of biomimetic hydrogel cell carriers with optimal printability and functionality hinder rapid advances in extrusion bioprinting. In fact, there is a significant gap between technology readiness level (TRL) 8–9 reached by bioprinters and TRL 3–4 for bioinks, highlighting the urgent need for bioink research [4,5]. Designing bioinks for optimal cell function not only involves creating an adequate scaffold support with RGD domains (arginine–glycine–aspartic acid domains) for cell adhesion [6] but also providing complex molecular pools representing healing environments [7] and triggering cell activities.

Recently, the use of blood-derived products, such as platelet-rich plasma (PRP), has been expanded in clinical regenerative medicine, as their molecular complexity is necessary to address the pathophysiological needs involved in tissue healing [8]. Cubo et al. [9] pioneered the use of plasma for bioplotting. Since then, various plasma-based bioinks have been developed to functionalize bioplotted constructs [10]. Taymour et al. [11] and Ahlfeld et al. [12] used fresh frozen plasma (FFP) (i.e., platelet-poor plasma (PPP)) mixed with 3% alginate and 9% methylcellulose for bioinks, which were loaded with mesenchymal stromal cells (MSCs), and their target tissues were the liver and bone, respectively. On the other hand, Faramarzi et al. [13] created a printable biomaterial (ink) by blending 1% alginate with PRP to generate cardiovascular and musculoskeletal tissue constructs. According to the current consensus, we differentiate the terms “ink” and “bioink”, of which the latter refers to inks (printable biomaterials) formulated with cells (i.e., “bio”) [14].

To date, no study has compared bioinks based on PPP (also called FFP), which is mostly devoid of the platelet secretome, and PRP-based bioinks. Automated blood-bank systems produce both products from a single donation. In regenerative medicine, PRP can be obtained in a single step by centrifugation at low force (i.e., 300–600 g) for 8–10 min, while PPP is obtained as a secondary by-product after double spin or filtration, following in-house or commercial protocols. We hypothesized that these two different plasma formulations, PRP and PPP, which substantially differ because of the molecular cargo provided by the platelet secretome, would influence different healing mechanisms. In this study, we examined the advantages and disadvantages of cellular constructs manufactured with both products in parallel for targeted and personalized wound management.

By proposing plasma as a central ingredient in bioink development, we aim to take advantage of the fibrin scaffold formed upon plasma coagulation and subsequent presentation of growth factors and cytokines playing key roles in healing processes, including inflammation, angiogenesis, and tissue anabolism. The differences and similarities between PRP and PPP can be enhanced during bioink development. Although PRP contains supraphysiological concentrations of platelets (2–10-fold enrichment; baseline levels of 250 × 10^3^·μL^−1^) and, optionally, leukocytes, PPP has a platelet count far below that in peripheral blood (<20 × 10^3^ μL^−1^). Moreover, although PPP provides a pool of plasma proteins, PRP additionally supplies a pool of signaling proteins contained inside the α-granules of platelets, which are released after formation of the fibrin clot.

Although the final goal of bioprinting is to reproduce tissue and organ complexity and heterogeneity for clinical engraftment, we considered other medical needs that require less complexity of the bioprinted construct but a deeper knowledge of signaling proteins and healing mechanisms (i.e., creating advanced wound dressings with healing properties). The three main objectives of this study were, as follows: (1) explore the possibility of manufacturing advanced wound-dressing constructs by assessing the printability of blood-derived products combined with alginate (with and without dermal fibroblasts); (2) increase the range of functionalized inks suitable for bioplotting by comparing the two different plasma formulations using current printability assessment methodologies [15,16,17]; and (3) examine certain aspects of the construct functionality relevant to wound healing.

## 2. Materials and Methods

Bioink preparation: Two different plasma formulations were used to generate two different bioinks, a high-platelet-count PRP-based bioink and a low-platelet-count PPP-based bioink. PPP and PRP from anticoagulated (citrate phosphate dextrose anticoagulant) whole blood from six healthy donors were prepared by apheresis at a local blood bank (CVTJ, Galdakao Hospital, Bizkaia, Spain, CEIC n°. CES-BIOEF 201907). Briefly, peripheral blood was processed using an automated Reveos system (Terumo BCT, Inc., Lakewod, CO, USA). Platelets and leukocytes were counted in plasma preparations using a Beckman Coulter (Brea, CA, USA) counter. PPP and PRP were aliquoted and stored at −80 °C until use.

Freeze-thawed PPP and PRP were blended with alginic acid sodium salt (#180947; Sigma–Aldrich, St. Louis, MO, USA) prepared in prewarmed DMEM (previously autoclaved solution 8%, *w*/*v*). The inks were pre-crosslinked with 20 mmol CaCl_2_ (prepared in DMEM) and kept at 37 °C overnight prior to cell loading and bioprinting.

Rheological characterization: PRP/ALG, PPP/ALG, and pristine ALG samples were evaluated for rheological behaviors (Haake RheoStress 1 rheometer; Thermo Scientific, Vigo, Spain). Shear thinning was tested at constantly increasing shear rates from 0 to 100 s^−1^ using a serrated plate–plate geometry (d = 35 mm) with a gap distance of 1 mm at 22 °C. After an initial amplitude sweep test to detect the linear viscoelastic region between 0.01 Hz and 50 Hz, the elastic modulus (G′), viscous modulus (G″), and loss tangent (tanδ) were determined. Finally, a shear flow test was carried out in a shear rate range of 0.1 to 50 s^−1^ using the same probe and gap. Before starting the test, the samples were left for 3 min to allow their temperature to stabilize and residual stress to relax.

The obtained flow data were fitted to the Carreau–Yasuda model for shear-thinning materials (Equation (1)):

(1) $$\eta =\eta_{\infty }+\left(\eta_{0}-\eta_{\infty }\right){\left[1+{\left(\lambda_{c}\dot{\gamma }\right)}^{a}\right]}^{\frac{n-1}{a}},$$

where *η* is the viscosity, *η*_0_ is the zero-shear rate limit viscosity, *η*_∞_ is the infinite shear rate limit viscosity, *λ*_c_ is the relaxation time constant at a characteristic shear rate, *n* is the pseudoplasticity index, and *a* is the Carreau constant.

This model describes the behavior of fluids with the Newtonian plateau at a low (*η*_0_) and high (*η*_∞_) shear rate and a transition area governed by the parameters *n* and *a*. When the power law exponent is *a* > 0, *λ*_c_ is equal to the Carreau time constant. When *n* = 1, the model matches the linear Newtonian model. For fluidizing liquids (*n* < 1), the viscosity decreases with the increasing strain rate.

3D bioplotting process: We used a syringe-based extrusion 3D domoBIO printer prototype (Domotek, Gipuzkoa, Spain). The bioprinting parameters were as follows: material flow, 100%; speed, 3 mm·s^−1^; and layer height, 0.4 mm (Supporting Information, Table S1). The bioinks were dispensed through a 2.54-cm long, 20 G blunt needle (Drifton, Hvidovre, Denmark) attached to 5-mL luer-lock syringe (dicoNEX, ZARYS International, Zabrze, Poland). Circular disks were manufactured by filament deposition using a layer-by-layer technique with parallel alignment in each layer and an orientation shift of 90° between layers.

The printability of the biomaterial inks, including the assessment of extrudability under different bioplotting conditions and analyses of the filament shape and 3D scaffold fidelity, are described in the Supplementary Materials. To assess the spreading ratios, we divided the filament diameters by the internal needle diameter (0.6 mm).

Postprinting ionic crosslinking: After bioprinting, the constructs were immersed in a 100 mmol CaCl_2_ bath for 10 min at 37 °C. To examine the changes in shape, we compared print dimensions (height and diameter) before and after crosslinking using the ImageJ software (National Institutes of Health, Bethesda, MD, USA).

Construct stability: Crosslinked constructs (cellular and acellular) were deposited on 24-well plates, rinsed with DMEM/F12 Glutamax (Thermo Fisher Scientific, Waltham, MA, USA), and maintained under cell culture conditions in DMEM/F12 Glutamax supplemented with a 5% PR or 5% PP supernatant, i.e., the serum released after PRP or PPP clotting, respectively. These sera were used as medium supplements instead of fetal bovine serum (FBS) to meet cell requirements while avoiding interference from FBS. After manufacturing and at various time points (1, 4, 7, and 18 days), the scaffolds were removed, and their wet weights (*W*_t_) were recorded and compared to the initial wet weight (*W*_0_). To preserve sample integrity during construct handling, a customized trowel was 3D printed. To explore whether cells limited contacts between reacting groups via physical hindrance during crosslinking, we compared the stability of cellular and acellular constructs. A pristine ALG construct was used as a reference.

RITC-dextran release: To study the diffusion properties of 3D bioplotted constructs, 100 µg/mL RITC-dextran (70 kDa; R9379, Sigma–Aldrich) was added to the inks as a model molecule to mimic the diffusion of nutrients and waste products through the constructs. To study cumulative release profiles of the bioprinted constructs, the constructs were immersed in 600 µL of deionized water, which was collected and replenished at different time points (15, 30, 45, and 60 min and 24 and 96 h). As rhodamine-B emits red fluorescent light, the release profile was obtained by measuring the fluorescence using a plate reader (Varioskan^®^ Flash, Thermo Scientific; Ex. 544 nm, Em. 576 nm). The rhodamine-B fluorescent values obtained after crosslinking represented the initial point in the release profile. Sample concentrations were calculated using calibration curves prepared with known standards, and the cumulative release of RITC-dextran was determined.

Cell culture: Human adult dermal fibroblasts (NHDF-Ad) were purchased from Lonza (Basel, Switzerland). The cells were cultured between passages 4 and 9 in DMEM/F12 Glutamax supplemented with a 10% PR supernatant, 100 UI/mL penicillin, and 100 µg/mL streptomycin (Life Technologies, Carlsbad, CA, USA) at 37 °C in a humidified atmosphere with 5% CO_2_. When cells reached ~80% confluence, they were harvested using a trypsin–EDTA solution.

HUVECs (CRL-1730; ATCC, Manassas, VA, USA) were cultured up to passage 6 in endothelial cell growth medium-2 BulleKit (Lonza) supplemented with 10% PR supernatant, 100 UI/mL penicillin, and 100 µg/mL streptomycin (Life Technologies). The cells were maintained at 37 °C in a humidified atmosphere with 5% CO_2_. When cells reached ~80% confluence, they were harvested with trypsin–EDTA.

Scanning electron microscopy: Samples were washed three times for 5 min each with HEPES balanced salt solution (HBS; 15 mmol HEPES, pH 7.2, 120 mmol NaCl, 1 mmol CaCl_2_, 0.3 mmol MgCl_2_, and 4.15 mmol KCl). After the final wash, the samples were fixed with electron microscopy-grade formaldehyde at a final concentration of 4% in HBS for at least 2 h at room temperature. After fixative removal, the samples were washed three times with HBS for 5 min each and subjected to progressive dehydration with increasing ethanol concentrations (30%, 50%, 70%, 90%, 96%, absolute, and absolute; 30 min each) at room temperature. For complete sample dehydration, two incubations with hexamethyldisilazane were added for 20 min each. After thorough removal of hexamethyldisilazane, the samples were metalized and examined under a Hitachi S4800 (Tokyo, Japan) scanning electron microscope at the SGIker (UPV/EHU) microscopy core facility.

Total protein release: PPP/ALG and PRP/ALG scaffolds, with and without cells, were cultured in quadruplicate in 500 µL of DMEM/F12 Glutamax supplemented with 5% PP or 5% PR releasates, respectively, with 50 µL of the medium added every 7 days. After 1 h, 4 days, and 18 days, CM containing released proteins were collected and centrifuged for 8 min at 10,000× *g* to remove cell debris and alginate pieces. Total protein concentrations were measured in CM over time using the Pierce bicinchoninic acid (BCA) assay (Thermo Fisher Scientific). To determine the specific protein content released from the scaffolds, the BCA measurements of fresh media (time point 0 h) were subtracted from those for each time point.

In addition, acellular and cellular constructs were dissolved in citrate buffer (55 mmol Na-citrate, 22 mmol EDTA, and 150 mmol NaCl) for 20 min at 37 °C. Then, the total protein content of just printed dressings, and crosslinked dressings was measured to determine the protein loss during the manufacturing process.

Cell viability/distribution within bioprinted constructs: Bioinks were loaded with primary human dermal fibroblasts at a concentration of 1.5 × 10^6^ cells·mL^−1^ 2 h before bioprinting. After crosslinking, PRP and PPP bioprinted scaffolds were cultured in DMEM supplemented with a 5% PR or 5% PP supernatant, respectively, for up to 18 days.

The bioprinted scaffolds were examined using the LIVE/DEAD test kit (L3224; Thermo Fisher Scientific) with calcein AM for live cells and EthD-1 for dead cells. For the test, the scaffolds were washed three times for 5 min each and then placed in a staining solution (2 µmol calcein and 4 µmol ethD-1 in DMEM) for 20 min at 37 °C in a 5% CO_2_ atmosphere. Fluorescence readings were carried out on a Synergy HT plate reader (BioTek, Winooski, VT, USA). Measurements of acellular scaffolds were used to subtract the signals generated by the PPP and PRP inks from those of cellular samples/constructs. Immediately after plate readings, the calcein and Ethd-1 signals from the matrix-embedded cells were visually inspected, and micrographs of three representative fields were obtained using an inverted fluorescence microscope with a 4× objective. The calcein/EthD-1 ratio was used as an index of cell survival.

Flow cytometry of live and dead cells: Cell viability was measured by flow cytometry using the LIVE/DEAD cytotoxicity test for mammalian cells (Thermo Fisher). Scaffolds were dissolved in citrate buffer (55 mM Na-citrate, 22 mmol EDTA, and 150 mmol NaCl, pH 7.2) with rotational incubation for 20 min [18]. Once a scaffold was completely dissolved, the cell suspension was filtered through a 70-μm pore diameter cell strainer and pelleted in a swinging bucket centrifuge for 5 min at 300× *g*. Cells were resuspended in a staining solution (PBS containing 0.1 µmol calcein and 1 µmol EthD-1), and after a 20-min incubation at room temperature in the dark, the samples were analyzed by flow cytometry (Gallios; Beckman Coulter). Before sample analysis, photomultipliers and gates were adjusted using unlabeled cells (as a negative control), healthy NH3T3 cultured cells (for the calcein signal as an alive control), and ethanol-fixed NH3T3 cells (for the EthD-1 signal as a dead control).

Protein precipitation for Western blot: Scaffolds were dissolved in citrate buffer as for cell viability measurements. Three scaffolds were dissolved together per replicate. Once completely dissolved, cell suspension was pelleted at 1000× *g* in a swinging bucket rotor for 5 min at 4 °C. After careful supernatant removal, cell pellet was resuspended in ice-cold lysis buffer (25 mmol Tris-Cl pH 7.5; 150 mmol NaCl; 1% SDS; 1% TX100) and homogenized through sonication in ice (three 20 s bursts of 90% amplitude with 1 min resting periods between each other), using a Bandelin Sonoplus mini20 ultrasonic homogenizer fitted with an MS1.5 probe (Bandelin electronic GmbH & Co., Berlin, Germany). Samples were cleared by centrifugation at 20,000× *g* for 10 min at 4 °C. Supernatants were stored at −20 °C before BCA protein quantification. After protein quantification, proteins were pelleted using the deoxycholate-trichloroacetic acid (DOC-TCA) method [19], resuspended in SDS-loading buffer, and heated at 100 °C during 5 min.

Whole cell lysates of NIH3T3 murine embryonic fibroblasts and CT5.3 human colon carcinoma-associated fibroblasts were used as positive controls for fibroblast and activated fibroblast markers, respectively.

Western blot: A total of 10 µg of protein were loaded in each lane (15 combs) of a 1 mm minigel of 4%–20% PAGE and run in running buffer (25 mmol Tris pH 8.3; 192 mmol glycine; 3.5 mmol SDS). Proteins were transferred to nitrocellulose membranes in transfer buffer (25 mmol Tris pH 8.3; 192 mmol glycine; 20% methanol; 3.5 mmol SDS) during 3 h in a cold room at a constant current intensity of 400 mA and variable voltage (250 V max) and power (600 W max). Membrane blocking was done with 5% nonfat-dry milk dissolved in TBST (10 mmol Tris-HCl pH 8;150 mmol NaCl; 0.05% Tween20). Appropriate antibody incubation was done overnight in blocking solution in a cold room with permanent agitation. Secondary antibodies were incubated in TBST for 2 h at room temperature in constant agitation, washed 3 times in TBST and developed using the Immobilon Crescendo Western HRP Substrate (WBLUR0500 Millipore, Burlington, MA, USA). Western blotting images were taken in a Syngene G box imaging system (Syngene, Bangalore, India).

ELISA of VEGF, PDGF-BB, RANTES, MCP-1, IL-8, ECM-1, PF4, TGF-β, HGF, bFGF, IGF-1, and FN: To examine whether acellular constructs released signaling molecules to the environment, VEGF, PDGF-BB, RANTES, MCP-1, PF4, IGF-1, and FN were measured in CM at different time points. To further determine whether plasma-functionalized bioinks influenced the cellular behavior after construct manufacturing, the VEGF, PDGF-BB, RANTES, MCP-1, IL-8, ECM-1, PF4, TGF-β, HGF, bFGF, IGF, and FN concentrations were assessed in CM of cellular constructs at 1 h, 4 days, and 18 days. The following ELISA kits have been used following the manufacturer’s instructions: human VEGF standard ABTS ELISA development kit (sensitivity: 16 pg/mL; 900-K10, Peprotech, Inc., Rocky Hill, NJ, USA); human PDGF-BB standard ABTS ELISA development kit (sensitivity: 16 pg/mL; 900-K04, Peprotech, Inc.); human RANTES (CCL5) standard ABTS ELISA development kit (sensitivity: 16 pg/mL; 900-K33, Peprotech, Inc.); human MCP-1 (CCL2) standard ABTS ELISA development kit (sensitivity: 8 pg/mL; 900-K31, Peprotech, Inc.); human IL-8 (CXCL8) Standard ABTS ELISA Development Kit (sensitivity: 8 pg/mL; 900-K18, Peprotech Inc.); human ECM1 ELISA Kit (sensitivity: 36 pg/mL; ab246524, Abcam, Cambridge, United Kingdom); human PF4 ELISA kit (sensitivity: 20 pg/mL; ab100628, Abcam); human TGF beta 1 ELISA kit (sensitivity: 80 pg/mL; ab100647, Abcam); human HGF ELISA (sensitivity: 3 pg/mL; ELH-HGF-1, RayBiotech Life Inc., Peachtree Corners, GA, USA); human bFGF ELISA (sensitivity: 50 pg/mL; ELH-bFGF, RayBiotech); human IGF-1 ELISA (sensitivity: 100 pg/mL; ELH-IGF1, RayBiotech); human Fibronectin ELISA (sensitivity: 0.6 ng/mL; ELH-FN1, RayBiotech). The experiments were performed in quadruplicate.

Cell proliferation: The proliferation of HUVECs incubated in the CM collected from PRP and PPP bioprinted scaffolds (1 h, 4 days, and 18 days) was evaluated. After collection, the cell media were centrifuged 8 min at 9600× *g* and then filtered through spin centrifuge filters (0.45-µm pore). Each conditioned media was diluted 1:1 with EGM-2 endothelial cell growth medium (Lonza). Before the experiment, the cells were starved in serum-free media for 24 h. Then, 1 × 10^5^ and 3 × 10^4^ cells·mL^−1^ were seeded into 96-well plates, and the plates were incubated for 24 h and 7 days, respectively. The XTT solution (Sigma–Aldrich) was added to the cells, and the incubation was continued for 4 h. The absorbance of each well was measured at a wavelength of 490 nm.

Wound healing assay: HUVECs were seeded into 96-well plates to create a confluent monolayer. The cells were starved for 24 h, and the monolayer was scraped with a 200-µL pipette tip to create a wound. The culture media were removed to discard the cell debris and then replaced with collected CM from PRP and PPP bioprinted scaffolds (1 h, 4 days, and 18 days). At the 0 h and 24 h time points, photographs of the same field of each well were acquired. To quantify wound closure, the area of each wound (0 and 24 h) was measured using the ImageJ software.

Statistical analysis: Data are expressed as the mean ± SD, unless otherwise specified. Assays were performed in quadruplicate for the six matched PRP and PPP donors; each donor was considered an independent sample. For more than two groups at one time point, a one-way ANOVA was performed. Repeated-measures ANOVA was performed for multiple time points. Non-normal data were examined using the Friedman test, followed by Wilcoxon paired comparisons. Correlations were calculated using the Pearson/Spearman coefficient. For all comparisons, statistical significance was set at *p* ≤ 0.05. Data were analyzed using SPSS for Windows version 18.0 (SPSS, Inc., Chicago, IL, USA).

## 3. Results

### 3.1. Composite Plasma Bioinks

#### 3.1.1. Individual Components of the Printable Biomaterials

We evaluated two different plasma composites based on PRP and PPP blends with alginate (PRP/ALG and PPP/ALG) to compare their physicochemical and functional features. The cellular components present in PRP and PPP are shown in Table 1. To obtain meaningful and reproducible results, we independently prepared bioinks with PRP and PPP from six donors, including two men and four women with a median age of 52.5 years (range: 19–60 years), and the following ABO groups/Rh types: A + (*n* = 5) and O + (*n* = 1).

#### 3.1.2. Ultrastructure and Rheological Properties of the Composite Biomaterials (Inks)

**Figure 1:**
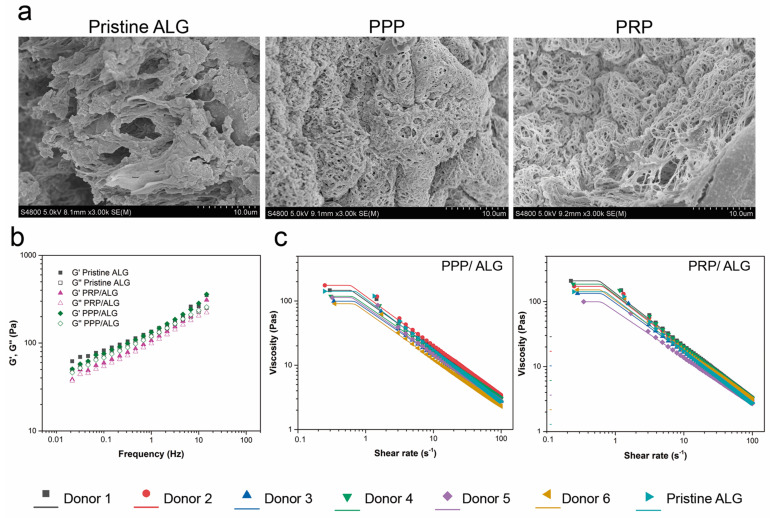
Ultrastructural and rheological properties of pristine ALG, PPP/ALG and PRP/ALG inks. (**a**) Separate ultrastructural scans of the alginate, PRP and PPP hydrogels used to formulate PPP/ALG and PRP/ALG bioinks. Original magnification, 3.00× k. Scale bar, 10 µm. (**b**) Storage and loss moduli measured by frequency sweep tests of pristine ALG, PRP/ALG, and PPP/ALG samples for six donors. (**c**) Flow curves of PRP/ALG and PPP/ALG samples and the theoretical Carreau–Yasuda model for six donors. ALG, alginate; PPP, platelet-poor plasma; PRP, platelet-rich plasma.

The individual components of the inks show different ultrastructure, as shown in Figure 1a. Although alginate exhibits interconnected sheets forming a compact structure, Ca^2+^-activated PRP and PPP hydrogel micrographs show a tight fibrin network with high porosity.

Strain-sweep tests were performed at a constant frequency of 1 Hz. Based on the values obtained, stress–strain curves were generated (data not shown) and used to obtain the limit strain values in the linear viscoelastic range for use in frequency sweep tests. The results are shown in Figure 1. All samples showed an elastic behavior, because the storage modulus (G′) was greater than the loss modulus (G″) in the studied interval, and low dependence on the frequency was observed in the system. Additionally, the overall viscoelasticity of the system can be described by loss-tangent values. Therefore, for a material in a gel-like state, depending on the gel strength, the loss tangent becomes lower than unity (0.82 to 0.92 in this study). Similarly, the dough-printability can be assessed in terms of the minimum pressure required and modeled using the loss tangent. Furthermore, the results of analyzing the flow behavior of the samples and the dependence of apparent viscosities on the shear rate are shown in Figure 1. All samples displayed similar steady-state viscosity (*η*) patterns of non-Newtonian fluids, with a shear-thinning behavior and a tendency to reach a Newtonian plateau zone at a low shear rate; thus, zero-shear viscosity (*η*_0_) was estimated.

The obtained experimental flow data were satisfactorily fitted to the Carreau–Yasuda model, with the resultant fitting parameters are listed in Table 2. All samples showed relatively low flow index values (*n* < 0.29), corroborating the shear-thinning behavior of the samples. Tee PRP/ALG and PPP/ALG blends met the rheological conditions for bioprinting without significant inter-individual differences.

#### 3.1.3. Printability Outcomes

To assess PRP/ALG and PPP/ALG ink capabilities for extrusion and formation of reproducible and stable scaffolds, we examined differences in printability parameters and outcomes of both bioink modalities. Table S1 shows the systematic variation of bioprinter parameters used to set up the manufacturing process, and Figure S1 shows the flowchart of the assays performed to analyze the printing outcomes. The spreading ratios significantly differed for the PRP/ALG and PPP/ALG inks (2.10 ± 0.40 vs. 1.70 ± 0.39, respectively; *p* < 0.001), which was confirmed by the differences between the areas of the PRP/ALG and PPP/ALG filament prints (54.04 ± 6.23 vs. 45.01 ± 5.64 mm^2^, respectively; *p* < 0.001). There were no significant inter-donor differences within the PPP/ALG (*p* = 0.502) and PRP/ALG (*p* = 1.000) ink groups.

Postprinting hydrogel polymerization with 100 mmol CaCl_2_ at 37 °C for 10 min induced significant changes in the dimensions of the discs for both bioink modalities. The areas of the base of the discs underwent a similar 15% reduction (from 80.1 ± 3.5 to 68.2 ± 4.2 mm^2^ for PRP/ALG and from 80.1 ± 3.5 to 68.1 ± 3.27 mm^2^ for PPP/ALG; both *p* = 0.028). Moreover, there were 19% and 17% increases in the heights of the constructs (from 1.77 ± 0.14 to 2.11 ± 0.17 mm for PRP/ALG and from 1.81 ± 0.10 to 2.12 ± 0.12 mm for PPP/ALG; both *p* = 0.028). Pristine ALG constructs without plasma underwent a 2.5% area retraction (from 76.2 ± 3.4 to 70.8 ± 6.2 mm^2^, *p* = 0.011), with a parallel 9.29% increase in the height of the discs (from 1.83± 0.06 to 2.00 ± 0.19 mm, *p* = 0.046).

### 3.2. Properties of Bioprinted Constructs

**Figure 2:**
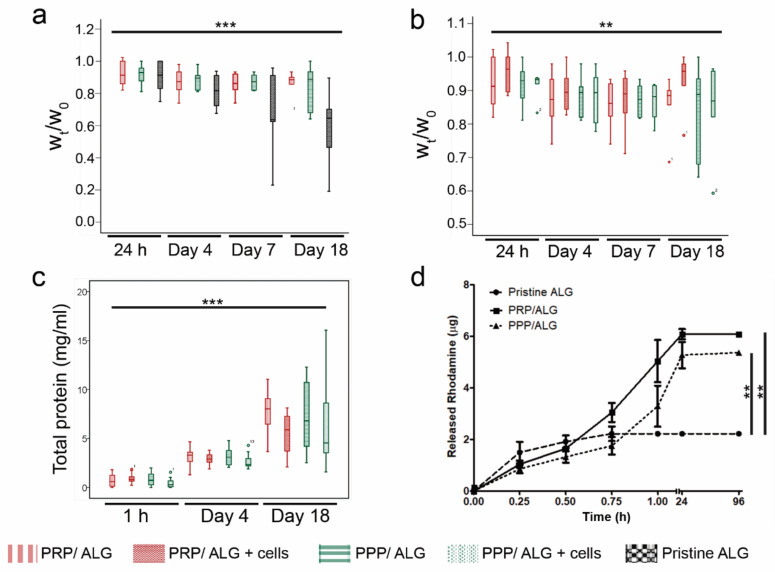
Properties of acellular and cellular dressings. (**a**) Box plots show changes over time of the median and 25th and 75th percentiles of the *W*_t_/*W*_0_ ratio of acellular PRP/ALG, PPP/ALG and pristine ALG constructs, where *W*_0_ is the initial postprinting weight, and *W*_t_ is the construct weight at time *t* (24 h and days 4, 7, and 18). (**b**) Box plots show the median and 25th and 75th percentiles of the *W*_t_/*W*_0_ ratio of cellular and acellular constructs. (**c**) Total protein release from cellular and acellular constructs over time (1 h and days 4 and 18). (**d**) Patterns of RITC-dextran release by different inks. ** *p* < 0.01; *** *p* < 0.001. ALG, alginate; PPP, platelet-poor plasma; PRP, platelet-rich plasma; *W_t_*, weight at time *t*; *W*_0_*,* weight at time 0; RITC, Rhodamine-B-isothiocyanate.

Cellular and acellular constructs were examined in terms of their in vitro stability over time and the flow of total protein release during manufacturing and thereafter (Figure 2). In an ANOVA with repeated measurements, acellular PPP/ALG, PRP/ALG, and pristine ALG constructs showed a significant decrease in stability over time (*p* < 0.001) and without significant influence of the biomaterial ink type (*p* = 0.055) (Figure 2a). However, during manipulation, the pristine ALG constructs were more fragile than the PRP/ALG and PPP/ALG scaffolds. After 18 days, 91.7% of the PRP/ALG and PPP/ALG constructs were intact as compared with 61.5% of intact ALG constructs.

We then determined whether cells could interfere with the crosslinking by limiting contacts between reacting groups via physical hindrance. Overall there was a significant decrease in stability (*p* = 0.006), but the presence of cells had no impact on the stability of the constructs (*p* = 0.969) (Figure 2b).

To confirm that there were no losses of plasma protein during the manufacturing process, we assessed the total protein retained within the scaffolds via their dissolution in citrate buffer immediately after the entire manufacturing process, which included bioprinting and subsequent cross-linking. Overall, there was a marginal loss of protein during the procedure, with 86.1% of the total protein remaining within the cross-linked scaffolds.

Both PRP/ALG and PPP/ALG cellular and acellular dressings showed similar temporal patterns of protein release into conditioned media (CM) (*p* < 0.001) without a significant interaction between time and ink/bioink type (*p* = 0.14) (Figure 2c). Thus, there were no differences in the protein-release pattern between cellular and acellular scaffolds (Figure 2c). The release of rhodamine B-isothiocyanate (RITC)-dextran (Figure 2d) confirmed the nutrient- and waste-diffusion capabilities of the constructs, with the PRP/ALG and PPP/ALG constructs showing higher release of rhodamine than pristine ALG (*p* < 0.003 and *p* = 0.001, respectively) at 24 h. During cross-linking, RITC-dextran shrinkage was observed in all constructs (27.82% in pristine ALG, 17.03% in PRP/ALG, and 9.96% in PPP/ALG).

**Figure 3:**
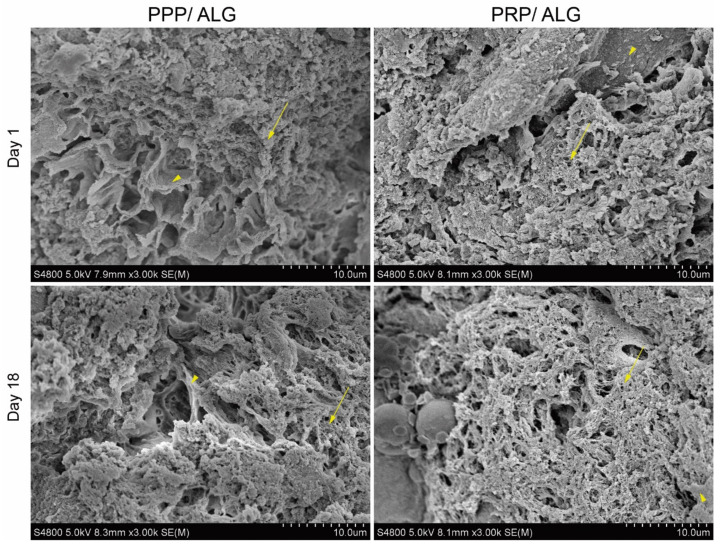
Scanning electron microscopy micrographs of PPP/ALG and PRP/ALG advanced dressings taken on days 1 and 18 following bioprinting. Fibrin fibers are indicated by arrows, and alginate is indicated by arrowheads. Original magnification, 3.00× k. Scale bar, 10 µm. PPP, platelet-poor plasma; PRP, platelet-rich plasma; ALG, alginate.

The ultrastructures of the PPP/ALG and PRP/ALG dressings showed a cavernous and heterogeneous nature, with two well-differentiated textures observed (Figure 3). Both alginate sheets and fibrin fibers proved to be robust enough to last for up to 18 days after printing. Overall, these results revealed that plasma hydrogels combined with alginate were stable over a period of 18 days and maintained their ultrastructure over time. In accordance with scanning electron microscopy (SEM) images, the PRP/ALG constructs showed the highest diffusion after 24 h and 96 h (6.08 ± 0.03 µg) as compared with PPP/ALG (5.36 ± 0.04 µg) and pristine ALG (2.22 ± 0.01 µg).

### 3.3. Cell Viability within PRP/ALG and PPP/ALG Bioprinted Constructs

**Figure 4:**
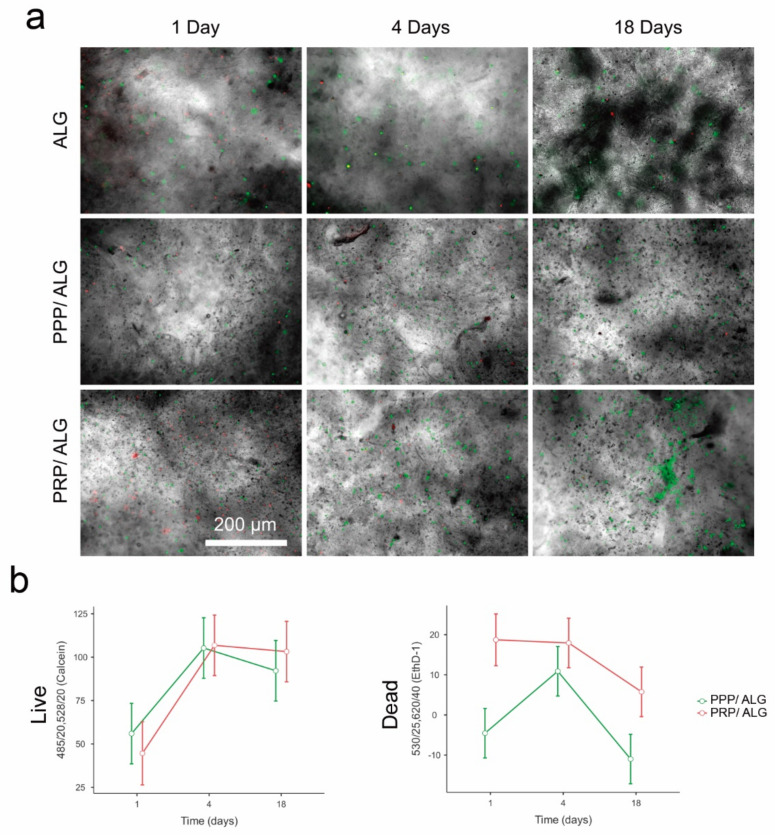
Live/dead assay results for ALG, PPP/ALG, and PRP/ALG dressings on days 1, 4, and 18 after bioprinting. (**a**) Fluorescence microscopy images of live (calcein = green) and dead (etdh-1 = red) cells. Original magnification, 4×. Scale bar, 200 µm. (**b**) Fluorescence readouts (fluorescence arbitrary units) obtained for PPP/ALG (green) and PRP/ALG (red) cellular dressings minus the signal obtained for acellular scaffolds. The graph in the left represents the fluorescent signal of calcein living cells, and the graph in the right represents the fluorescence emitted by etdh-1 in dead cells. ALG, alginate; PPP, platelet-poor plasma; PRP, platelet-rich plasma.

During scaffold manufacturing, cells embedded within a hydrogel experience stressful signals that occur with changes in temperature and strain forces associated with filament extrusion through the nozzle. We performed live/dead cell-viability assays and analyzed the bioprinted constructs over 18 days by fluorescence microscopy and fluorescence readings using a plate reader (Figure 4). Cell death was mainly observed within the first 4 days after bioprinting, and progressive decay was observed between days 4 and 18. Remarkably, unlike the acellular PPP/ALG constructs, the acellular PRP/ALG constructs displayed an intense ethidium homodimer 1 (ethD-1) signal, which was attributed to the leukocyte debris generated during the freeze/thaw procedure of PRP production (Figure 4b). Moreover, calcein fluorescence increased considerably during the first 4 days in both bioinks, suggesting metabolic recovery of cells after the stress exerted by the bioprinting procedure, and the fluorescence was sustained until the end of the experiment. Notably, at 18 days, PRP/ALG dressings exhibited higher calcein fluorescence signals than PPP/ALG dressings, which correlated with a higher cell density observed in the PRP/ALG constructs according to fluorescence microscopy. Additionally, the homogeneous and spherical cell morphology displayed in both cellular constructs in the early days transformed into an elongated morphology and the formation of small and healthy colonies at 18 days, predominantly in the PRP/ALG dressings.

**Figure 5:**
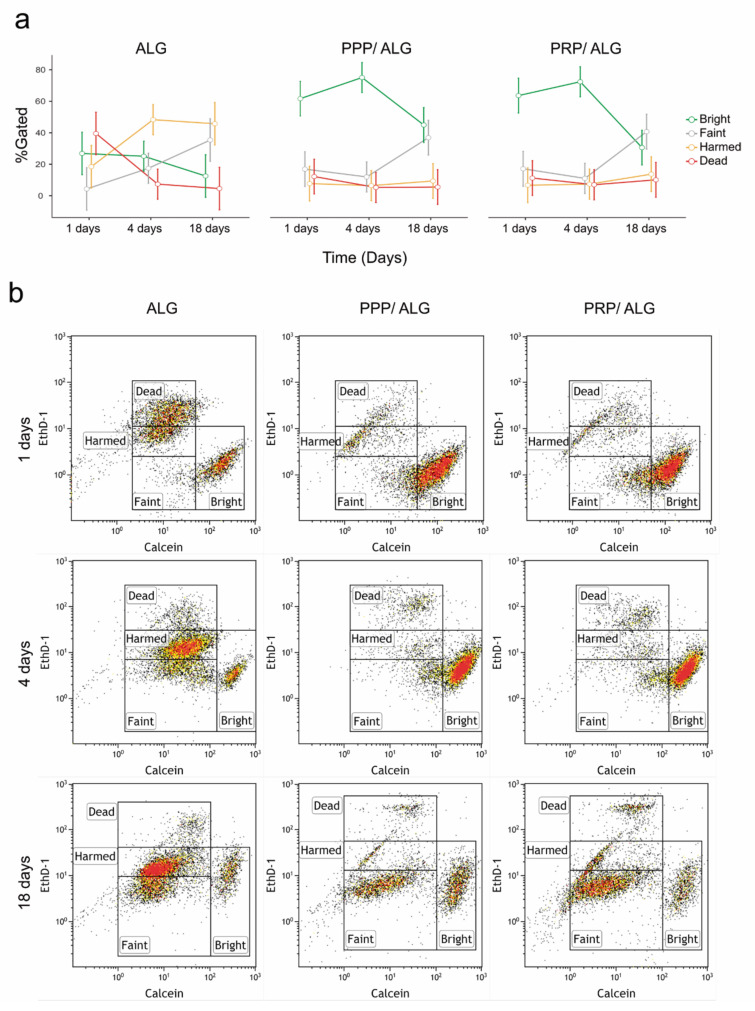
Live/dead flow cytometry analysis of diluted ALG, PPP/ALG, and PRP/ALG dressings on days 1, 4, and 18 after bioprinting. (**a**) Four cell populations were observed based on calcein and eth-D1 fluorescence signals: dead, injured, bright (alive with high metabolic activity), and faint (alive with low metabolic activity). (**b**) Flow cytometry density plots corresponding to 1, 4, and 18 days after bioprinting and showing the dead, injured, bright, and faint cell populations. ALG, alginate; PPP, platelet-poor plasma; PRP, platelet-rich plasma; etdh-1, ethidium homodimer-1.

To further investigate the viability and functional activity of cells in the bioprinted scaffolds, we performed live/dead flow cytometry after scaffold dissolution in citrate/EDTA. Fluorescence microscopy and flow cytometry analyses performed in parallel have not been reported previously. Interestingly, the latter provided more accurate information by revealing different populations of embedded cells according to the calcein and ethD-1 fluorescence signals (Figure 5). The former is directly proportional to the intracellular esterase activity of live cells. In this regard, we were able to detect at least two different cell populations in terms of their esterase activity: one with a bright signal and another with a faint one, thus representing more and less metabolically active cell populations, respectively (Figure 5). The faint population was never observed on the day after bioprinting and required at least 4 days for its detection. Interestingly, cells embedded in the pristine ALG construct were more vulnerable to the stress caused by the bioprinting protocol, as indicated by a more representative dead-cell population throughout the experiment than in the PPP/ALG and PRP/ALG dressings.

**Figure 6:**
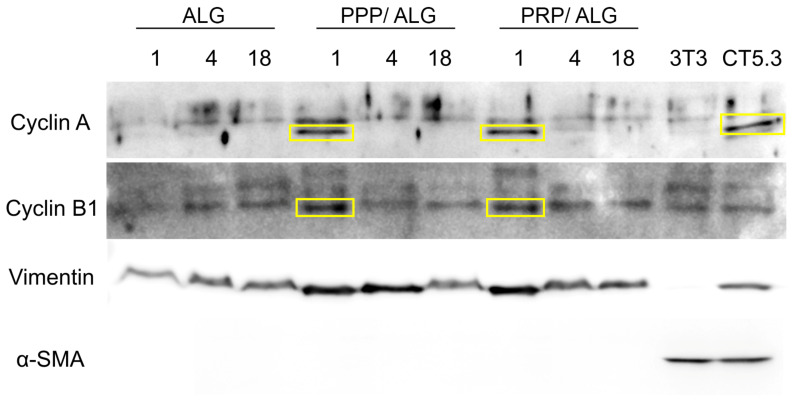
Western blot analysis of protein expression in pristine ALG-, PPP/ALG-, and PRP/ALG-dressing-embedded dermal fibroblasts over time (days 1, 4, and 18). Analysis of the expression of the proliferation markers cyclins A and B1 and the cytoskeletal proteins vimentin and α-SMA. An equal amount of protein was loaded in each lane. Protein extracts of NIH3T3 fibroblasts and CT5.3 colon cancer-associated fibroblasts were used as controls. ALG, alginate; PPP, platelet-poor plasma; PRP, platelet-rich plasma; α-SMA, smooth muscle α-actin.

Based on these results, we investigated the expression of proliferation markers and fibroblast cytoskeletal components, such as vimentin and α-smooth muscle actin (α-SMA), as markers of fibroblast activation in the scaffold resident fibroblasts. Protein-expression profiles suggested that the proliferation markers cyclin A and cyclin B1 were only expressed when the bioink blend included either PPP or PRP, as these are both absent in pristine ALG (Figure 6). Similarly, decreases in both markers at 4 and 18 days after printing suggested that the culture underwent quiescence, thereby supporting the cell-viability and -functionality results obtained previously (Figure 5).

In regard to cytoskeletal markers, vimentin demonstrated stable expression during the 18 days in pristine ALG (Figure 6), whereas the addition of plasma to the formulation initially increased vimentin levels relative to those in pristine ALG before dropping again after 18 days. Additionally, we detected no α-SMA in any dressing, with this exclusively observed in positive controls (NIH3T3 embryonic fibroblasts and CT5.3 colon tumor-associated fibroblasts). Remarkably, vimentin was not detected in embryonic murine NIH3T3 fibroblasts. The employed vimentin antibody was not designed to recognize murine vimentin, which is indicative of the veracity of the vimentin signal on the rest of the lanes. On the contrary, α-SMA antibody is predicted to recognize murine α-actin, which is widely expressed due to the FBS-induced fibroblast activation during cell culture.

### 3.4. Functionalities of Bioprinted Advanced Dressings

PRP and PPP contain variable amounts of regulated upon activation, normal T cell expressed and presumably secreted (RANTES), insulin-like growth factor 1 (IGF-I), transforming growth factor beta (TGF-β), platelet factor 4 (PF4), monocyte chemotactic protein 1 (MCP-1), basic fibroblast growth factor (bFGF), hepatocyte growth factor (HGF), and platelet-derived growth factor (PDGF). There were no passive releases of either growth factors [PDGF-BB, IGF-1, vascular endothelial growth factor (VEGF), HGF, TGF-β1, and bFGF], cytokines (PF4, MCP-1, and RANTES), or extracellular matrix proteins [extracellular matrix protein 1 (ECM-1) and fibronectin (FN)] in the PRP/ALG and PPP/ALG acellular constructs (data not shown). Remarkably, the plasma/alginate constructs retained growth factors and chemokines over time.

#### 3.4.1. De Novo Synthesis and Release of VEGF, MCP-1, HGF, Interleukin (IL)-8, FN, and ECM-1

**Figure 7:**
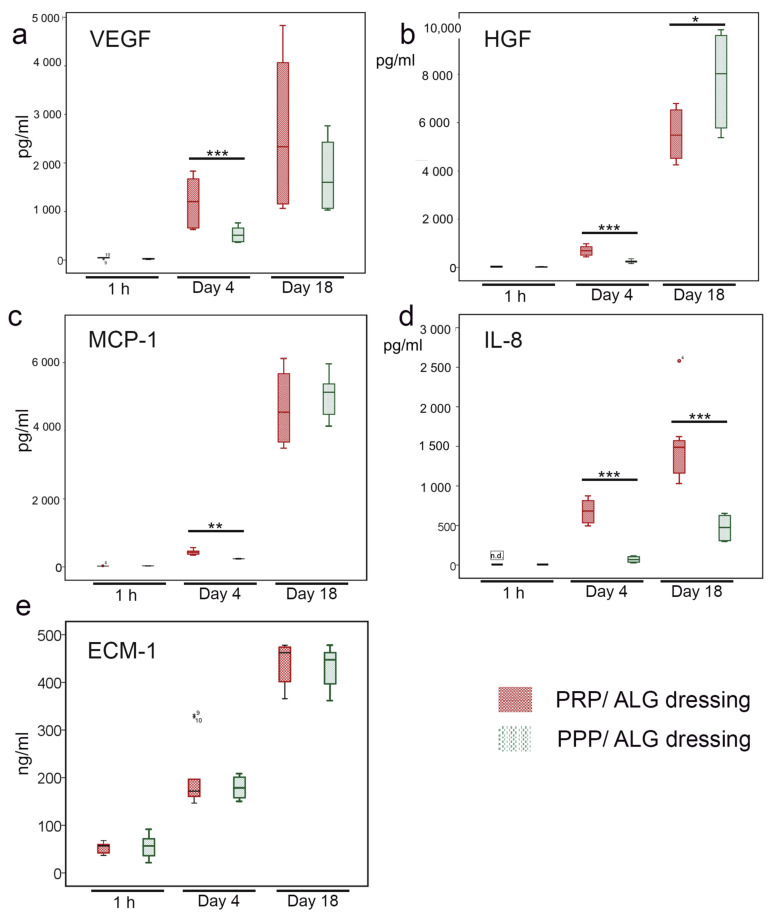
Production/Release of angiogenic and immune modulators by PRP/ALG and PPP/ALG bioprinted dressings at the indicated time points. Box plots showing changes over time (1 h, day 4 and 18) of the median and 25th and 75th percentiles of the released levels of (**a**) VEGF (pg/mL), (**b**) HGF (pg/mL), (**c**) MCP-1 (pg/mL), (**d**) IL-8 (pg/mL), and (**e**) ECM-1 (ng/mL). * *p* < 0.05; ** *p* < 0.01; *** *p* < 0.001. ALG, alginate; PRP, platelet-rich plasma; PPP, platelet-poor plasma; VEGF, vascular endothelial growth factor; HGF, hepatocyte growth factor; MCP-1, monocyte chemotactic protein 1; IL-8, interleukin-8; ECM-1, extracellular matrix protein 1.

Functionalized advanced dressings were designed to modify the cytokine balance in the pathological angiogenic and inflammatory wound microenvironment. Both PPP/ALG and PRP/ALG dressings released VEGF over time, and at 4 days, levels of VEGF were significantly higher in the PRP/ALG dressings than in the PPP/ALG dressings (*p* < 0.001), although the difference was not significant at 18 days (Figure 7a). HGF was also synthesized/released by the PPP/ALG and PRP/ALG dressings. Although the PRP/ALG dressings released more HGF at 4 days (*p* < 0.001), after 18 days, HGF levels were higher in the PPP/ALG dressings (*p* = 0.038), representing a 31-fold increase versus an 8-fold increase in PRP/ALG relative to the concentrations on day 4 (Figure 7b). A significant progressive release of MCP-1 from the PRP/ALG dressings was also observed over time (*p* = 0.002). The release began on day 4 (437.3 ± 70.6 pg·mL^−1^) and reached 4674.5 ± 1071.4 pg·mL^−1^ on day 18, representing an 11-fold increase from days 4 to 18. Additionally, the PPP/ALG dressings showed significant synthesis of MCP-1 over time (*p* < 0.001). Although the initial increase was not as sharp as that in PRP/ALG (437.3 ± 70.6 vs. 241.0 ± 10.5 pg·mL^−1^ after 4 days, *p* = 0.002), the release of MCP-1 from the PPP/ALG scaffolds reached similar levels to those from PRP/ALG after 18 days (Figure 7c). Moreover, the PRP/ALG dressings showed a higher release of IL-8 than the PPP/ALG dressings on days 4 (*p* < 0.001) and 18 (*p* < 0.001) (Figure 7d), and both PRP/ALG and PPP/ALG dressings showed a similar sustained release of ECM-1 over time (*p* < 0.001) (Figure 7e). The synthesis/release of fibronectin by advanced PRP/ALG and PPP/ALG dressings was similar after 18 days. Although acellular PRP/ALG and PPP/ALG constructs released 22.13 ± 0.04 mg·mL^−1^ and 22.19 ± 0.76 mg·mL^−1^, respectively, levels in the CM from cellular constructs were 37.11 ± 1.78 mg·mL^−1^ for PRP/ALG and 34.62 ± 1.64 mg·mL^−1^ for PPP/ALG, respectively (*p* = 0.029 for both cellular and acellular constructs).

#### 3.4.2. Paracrine Effects on the Proliferation and Migration of Human Umbilical Vein Endothelial Cells (HUVECs)

**Figure 8:**
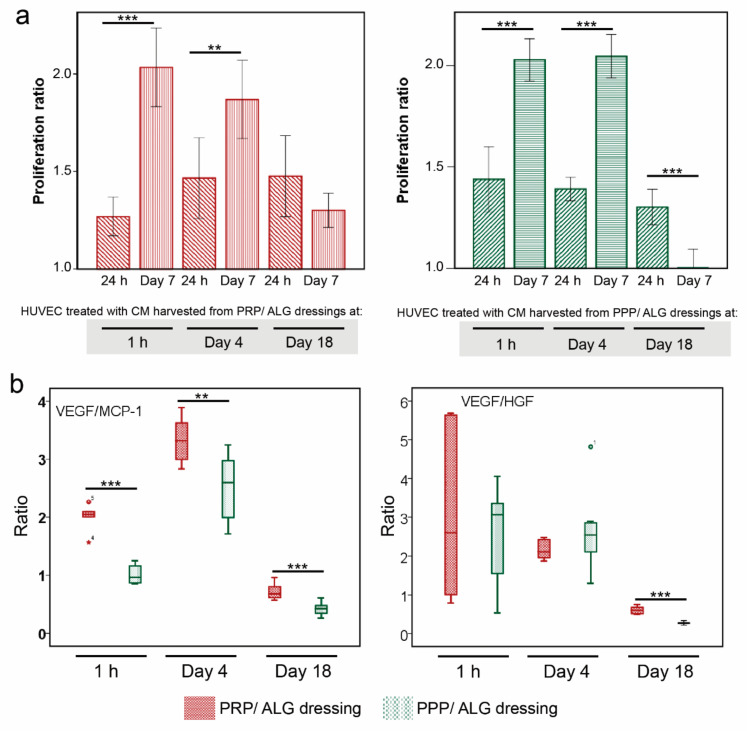
Effects of CM (1 h and days 4 and 18 after bioprinting) on HUVEC proliferation. (**a**) HUVEC-proliferation ratios after 24 h and 7 days of treatment with CM obtained from PRP/ALG and PPP/ALG dressings. OD values were normalized to those for control HUVECs, which were cultured under the same conditions in the absence of CM. (**b**) Box plots showing differences in the median and 25th and 75th percentiles of VEGF/MCP-1 (right) and VEGF/HGF (left) ratios between PRP/ALG and PPP/ALG dressings over time (1 h and days 4 and 18). ** *p* < 0.01; *** *p* < 0.001. CM, conditioned media; HUVEC, human umbilical vein endothelial cell; OD, optical density; ALG, alginate; PRP, platelet-rich plasma; PPP, platelet-poor plasma; VEGF, vascular endothelial growth factor; HGF, hepatocyte growth factor; MCP-1, monocyte chemotactic protein 1.

After 7 days of culture, endothelial cells proliferated similarly in culture media supplemented with 50% CM from both PRP/ALG and PPP/ALG dressings harvested at 1 h and 4 days. However, the proliferation decreased with the CM obtained 18 days after printing, particularly with that harvested from the PPP/ALG dressings (Figure 8a). These differences might be attributable to the composition of the conditioned media according to positive correlations between the VEGF/MCP-1 ratio and HUVEC proliferation (r = 0.434, *p* = 0.043 for PRP/ALG dressings; and r = 0.672, *p* < 0.001 for PPP/ALG dressings). Similarly, the VEGF/HGF ratio correlated with HUVEC proliferation (r = 0.411, *p* = 0.046 for PRP/ALG dressings; and r = 0.672, *p* < 0.001 for PPP/ALG dressings) (Figure 8b).

**Figure 9:**
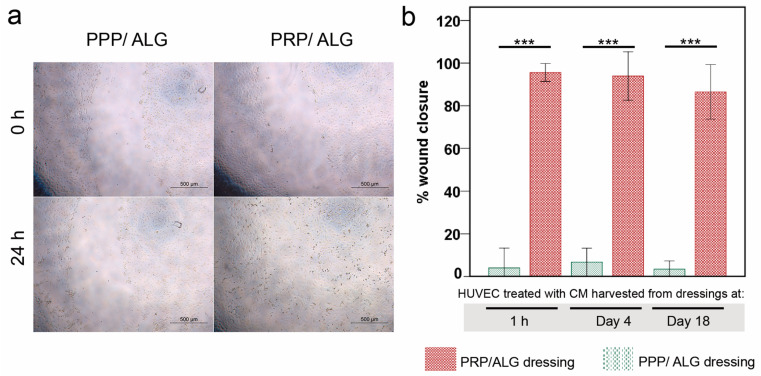
Scratch wound-healing assay on HUVECs treated with CM from PPP and PRP (1 h and days 4 and 18). (**a**) Photomicrographs illustrating differences in HUVEC migration at 0 h and after 24 h of exposure. CM. Original magnification, 10×. Scale bar, 500 µm. (**b**) Percentage of wound closure among HUVECs exposed to CM from PRP/ALG (red) and PPP/ALG (green) dressings harvested at 1 h, 4 days, and 18 days after bioprinting. *** *p* < 0.0001. CM, conditioned media; HUVEC, human umbilical vein endothelial cell; ALG, alginate; PRP, platelet-rich plasma; PPP, platelet-poor plasma.

The migration of vascular endothelial cells is important in vasculogenesis and angiogenesis. Here, the “scratch” wound-closure assay was used to assess the effect of CM on HUVEC migration. Wound closure was achieved after 24 h in the presence of CM obtained from PRP/ALG dressings. Overall, wound closure was similar with the CM obtained from all PRP/ALG dressings independent of harvest time. By contrast, HUVECs migrated poorly in the presence of CM obtained from PPP/ALG dressings at 1 h, 4 days, and 18 days (CM from PRP/ALG dressings vs. PPP/ALG dressings: 1 h, 23.85-fold increase; and 4 days, 13.32-fold increase and 18 days, 21.70-fold increase as compared with that observed with CM from PPP/ALG dressings. Therefore, the presence of platelets in the dressing is necessary for HUVEC migration into the wounded area (Figure 9).

## 4. Discussion

Bioprinting allows the reproducible manufacturing of advanced active dressings and tuning of their biological properties for personalized treatment. Because plasma alone does not meet the biomaterial requirements for extrusion [20], we prepared two bioinks by blending PRP and PPP with alginate and studied the printability outcomes, postprinting modifications, stability, and internal microarchitecture of two plasma-based inks (i.e., printable biomaterials) and their corresponding bioinks (i.e., cell-laden printable biomaterials) [14]. Additionally, rheological parameters were measured to ensure adequate blood plasma flow during the 3D printing process. Previous studies of the flow of formulated doughs through a nozzle demonstrate that the efficiency of the process is highly dependent on rheology [21]. In the present study, the network structure of the blends was close to collapse, because G′ and G″ were very close to each other, indicating a weak gel. Furthermore, no significant differences (*p* > 0.05) were observed in the behaviors of samples from different donors, with similar tan δ values [defined as the ratio between the loss and storage moduli (G″/G′)] obtained for all of the formulations.

Additionally, knowledge of viscosity is essential for designing the 3D-printing process, as well as for establishing flow conditions, such as syringe geometry or total printing time, among others [22]. The suitable viscosity for 3D printing should be low enough to permit extrusion through a nozzle but high enough for cohesiveness with the previously-deposited layer to maintain the shape. The shear-thinning performance, corroborated in this work by low flow index values (*n* < 0.29), indicated that the chains were affected by the shear stress between the layers when the flow rate increased, thereby reducing the force between them and a behavior that is reportedly beneficial for the 3D-printing process [22].

In previous clinical research, we investigated the efficacy of PRP therapies in the management of diabetic and vascular ulcers [23,24,25], and this knowledge inspired the development of plasma-based dressings for wound management. Acellular plasma bioprinted constructs contain a large pool of signaling proteins, which are not, however, passively released. This corroborates our previous research on wound management [26]. While investigating the most appropriate secondary dressings, we found that alginate (unlike other hydrocolloids or hydrofibers) demonstrated a high affinity for fibrin, and that a composite created thereof retained platelet-secreted PDGF-BB. Fibrin(-ogen) provides heparin-binding sites that enable the binding of specific growth factors, including VEGF-B, PDGF-AB, PDGF-BB, bFGF, TGF-β1, and brain-derived neurotrophic factor [27]. Furthermore, plasma fibronectin functions as a promiscuous growth factor-binding protein [28,29].

Cytokine retention is beneficial, as keeping plasma and the platelet secretome within the scaffold makes growth factors more accessible to embedded cells and influences cellular behavior after the structure is printed. Therefore, dermal fibroblasts were integrated within printable hydrogels to activate the cells with plasma molecules.

Precise determination of cell stress and viability is a major challenge to ascertain the quality of bioprinted advanced dressings. Moreover, it constitutes a bottleneck that hinders progress in bioprinting technologies and that has been overcome through the development of a reported innovative cell-monitoring protocol. In fact, by assessing postprinting cell viability within the constructs using conventional fluorescence microscopy and fluorometry, we found a cell subset whose state was questionable, especially in pristine ALG. Thus, cell viability determined by flow cytometry after fluidifying the hydrogel provided further insights into cell status. Here, we observed that two different populations of viable cells were distinguished according to their metabolic activity. The less active population increased in presence over time, with this explained by decreases in protein levels of cyclins A and B1, both associated with the G2 phase of the cell cycle. These results suggest that PRP- and PPP-dressing-embedded fibroblasts can survive for long periods without promoting exacerbated growth. The protective roles of PRP and PPP are supported by the low levels of cyclins in pristine-alginate-embedded cells and correlated with the increased presence of injured and non-proliferative fibroblasts observed under these conditions. Moreover, the functionalization of alginate with plasmas appeared to favor the construction of a more robust cytoskeleton, which could explain the increased survival of fibroblasts in the days immediately after printing. Neither formulation stimulated the appearance of α-actin, a known marker of fibroblast activation associated with fibrosis. These results supported the demonstrated functionality of the bioink without implying fibrotic activation of fibroblasts, which avoided the potential adverse effects associated with fibrosis during the healing processes.

Human skin heals mainly due to re-epithelialization and new dermis formation, with small wound contraction performed by the activated myofibroblasts [30]. This process is supported by the dressings produced in the present study, especially the PRP dressings. Here, we described the secretion of molecular factors that support angiogenesis, cell migration, and anabolic activities and in the absence of activation of resident fibroblasts, thereby demonstrating low levels of contraction.

Recently, Zhang et al. demonstrated the healing efficiency of PRP mixed with alginate polymerized with CaCl_2_ and thrombin in a porcine wound model [31]. However, this manually-manufactured acellular dressing was not dynamically active and required very frequent dressing changes (every 2 days). Cell inclusion within the dressings constitutes a major advantage, because they produce fresh neosynthesized molecules over time, thereby avoiding frequent dressing changes and subsequent nursing-time costs. Additionally, the automated 3D-bioprinted manufacturing of these dressings would enhance their reproducibility and allow personalization of the dressing-shape to the wound bed.

Ideally, according to the design principles for advanced wound dressings, sterile and biocompatible (neither cytotoxic nor immunogenic) hydrogels should be bioplotted and functionalized with cells blended with either autologous or allogeneic blood-derived products. These dressings should release healing molecules (modulators of angiogenic and immune responses) to the ulcer bed and be nonadhesive and easy to eliminate once they have fulfilled their function [32]. The plasma formulations that we selected to create our dressings (i.e., PPP and PRP) are prepared by apheresis in blood banks and have been used in transfusion medicine to treat coagulation factor deficiencies [33] or poor platelet function [34] respectively.

Platelets can tune the biological properties of advanced dressings, because they shape the inflammatory and immune responses by providing a complex pool of signaling proteins in the dressing, which will be available for embedded cells and thereby trigger an array of cellular reactions [35]. Advanced PRP dressings provide relevant concentrations of IL-8, a chemokine involved in avid recruitment of immune cells and the migration of endothelial cells [36]. Accordingly, because of the differences in platelet concentrations between PPP and PRP, significant differences were found in IL-8 production and the stimulation of endothelial cell migration between the respective dressings. Primarily, only PRP but not PPP dressings strongly promoted HUVEC migration, whereas both PRP and PPP dressings could induce HUVEC proliferation, irrespective of their maturation time in vitro.

Neovascularization is a prerequisite for tissue repair, because it provides blood supply, oxygen, and nutrients and facilitates infiltration of immune cells (monocyte/macrophage) into the wound. Typically, VEGF is the most prominent stimulator of endothelial cell proliferation. However, surprisingly, we found that CM harvested after 1 h and 4 days and with lower VEGF concentrations than those in CM obtained after 18 days had greater potential for promoting HUVEC proliferation. Thus, not only might temporal changes in VEGF expression regulate angiogenesis, but we also found that the VEGF/HGF and VEGF/MCP-1 ratios positively correlated with HUVEC proliferation. Interestingly, both PRP and PPP dressings produced relevant amounts of MCP-1 (CCL2), which is also involved in HUVEC apoptosis [35] and might explain the correlation between the VEGF/MCP-1 ratio and HUVEC proliferation. Furthermore, MCP-1 is a crucial pleiotropic chemokine that regulates monocyte/macrophage trafficking and infiltration into injured tissues [37]. Previous studies reported that CCL2-knockout mice show impaired immunomodulatory potential and reduced re-epithelialization, which was associated with reduced macrophage infiltration into the wound [38,39]. Moreover, the involvement of MCP-1 in macrophage repolarization was recently reported in an experimental model of CCL2-knockout mice [40]. Additionally, early diabetic wounds show decreased and impaired function, which were restored with one-time MCP-1 treatment [41].

The transition from the inflammatory to the proliferative phase is often compromised in chronic wounds. HGF (also known as scatter factor, SF) and its receptor c-MET are highly expressed in granulation tissue [42], and HGF neutralization is associated with delayed wound healing due to decreased granulation-tissue formation [43]. Therefore, recombinant HGF has been proposed for the treatment of diabetic foot ulcers [44]. Interestingly, we observed high concentrations of fibronectin produced and released by fibroblasts embedded within the scaffolds. The release of FN occurred in parallel with that of ECM-1, a less-investigated glycoprotein with an essential role in angiogenesis and wound-healing mechanisms [45].

The bioprinting of advanced dressings offers a possibility to select the time of construct maturation in vitro (e.g., 4–18 days) for a dynamic therapeutic based on paracrine interactions. Another opportunity could be extending the cell culture period and collecting CM for topical application once relevant concentrations of angiogenic factors have been reached. MSC-derived CM are currently in phase 1 clinical trials for chronic skin ulcers (NCT041346765), residual burn wounds (NCT04235296), and donor sites during skin grafting (NCT04234750).

The bioprinting process could be further optimized by improving bioink quality. In fact, we used a generic alginate instead of an optimized tailored alginate (i.e., with a higher guluronate (G):mannuronate (M) ratio, as only G blocks participation in cross-linking). Additionally, instead ethylene oxide treatments, we have sterilized alginate solutions by autoclaving, which is detrimental for alginate printability [46]. Both factors (low G:M ratio and alginate autoclaving) hinder optimal shape fidelity and are unfavorable for bioprinting outcomes. Moreover, different modalities of dynamic advanced dressings can be created by loading bioinks with mesenchymal stromal cells, which could offer additional advantages in avoidance of immune reactions.

Given our in vitro findings on the long-term stability and functionality of advanced active dressings, we envision further studies to obtain in vivo evidence on efficiently creating granulation tissue and responding to the hostile microenvironment of the pathologic wound bed.

## 5. Conclusions

This study shows the capabilities of 3D extrusion bioprinting for manufacturing functionalized constructs with adequate porosity for nutrient and waste diffusion, cell viability, and functionality. Moreover, our findings suggest that the advanced cell-laden dressings manufactured with plasma-based bioinks (both PRP and PPP) could provide complementary molecules with multifunctional roles in wound healing. Thus, these results together with the added advantages of bioprinting lay a foundation for developing further bioink modalities to tune biological properties of the wound microenvironment for expedited healing. These results indicated that the constructs behaved as advanced dressings with significant benefits for wound management, including angiogenic and immunomodulatory capacities and the synthesis and release of ECM molecules. Further in vivo studies are required to confirm the therapeutic potential of these dressings in wound management and clarify how often the advanced dressings should be replaced. Nevertheless, these findings offer insights into a novel concept of living, advanced dressings with tunable biological properties. Furthermore, the current clinical use of PRP, PPP, and alginate simplifies the implementation of the regulatory requirements mandatory for clinical translation and market access.

## Figures and Tables

**Table 1:** PRP and PPP cell components.

Component	PRP Median (Min–Max)	PPP Median (Min–Max)
Platelets × 10^3^·μL^−1^	2156 (1926–2316)	24 (15–36)
VPM (fL)	10.9 (9.6–11.7)	9.5 (8.6–13)
Platelet aggregates	229 (58–1698)	(24–57)
Leukocytes ×10^3^ [µL^−1^]	2.36 (0.65–16.6)	n.d.
Lymphocytes (%)	73.8 (68.5–83.3)	−
Monocytes (%)	9.85 (4.6–20)	−

PRP: Platelet-rich plasma; PPP: platelet-poor plasma; n.d.: not detected.

**Table 2:** Carreau–Yasuda model parameters and shear-rate values for plasma samples of six different donors.

Sample	*η*_0_ (Pa·s)	*a*	*n*	*λ* (s)	*R* ^2^
Pristine ALG	142	50.31	0.20	1.40	0.99
PRP/ALG (Donor 1)	209	34.36	0.19	1.71	1
PRP/ALG (Donor 2)	172	33.38	0.19	1.51	0.99
PRP/ALG (Donor 3)	133	24.25	0.24	1.56	0.99
PRP/ALG (Donor 4)	186	42.49	0.20	1.60	1
PRP/ALG (Donor 5)	100	24.58	0.29	1.60	0.99
PRP/ALG (Donor 6)	151	50.24	0.24	1.65	0.99
PPP/ALG (Donor 1)	147	31.01	0.25	1.62	0.99
PPP/ALG (Donor 2)	175	34.26	0.23	1.65	1
PPP/ALG (Donor 3)	100	32.28	0.28	1.46	0.99
PPP/ALG (Donor 4)	118	30.38	0.27	1.54	0.99
PPP/ALG (Donor 5)	113	33.59	0.27	1.58	0.99
PPP/ALG (Donor 6)	91	24.82	0.26	1.45	0.99

PRP: Platelet-rich plasma; PPP: platelet-poor plasma; ALG, alginate.

## Data Availability

The data presented in this study are available on request from the corresponding author.

## Supplementary Materials

The following are available online at https://www.mdpi.com/article/10.3390/biomedicines9081023/s1, Figure S1: Flowchart of the experiments performed to analyze printing outcomes., Table S1: Systematic variation in the bioprinter parameters used to establish the manufacturing process.

## Abbreviations

ALG	Alginate
bFGF	basic fibroblastic growth factor
CM	conditioned media
ECM-1	extracellular matrix-1
FN	Fibronectin
HGF	hepatocyte growth factor
HUVEC	human umbilical vein endothelial cells
IGF-1	insulin-like growth factor
IL-8	interleukin 8
MCP-1	monocyte chemotactic protein 1
PDGF-BB	platelet-derived growth factor-BB
PF4	platelet factor-4
PPP	platelet-poor plasma
PRP	platelet-rich plasma
RANTES	regulated upon activation, normal T cell expressed and presumably secreted
RITC	rhodamine-B-isothiocyanate
α-SMA	smooth muscle α-actin
TGF-β	transforming growth factor
VEGF	vascular endothelial growth factor
