# Supplementary material for: Plasma-Based Bioinks for Extrusion Bioprinting of Advanced Dressings
Source: Biomedicines. 2021 Aug 16;9(8):1023. doi: 10.3390/biomedicines9081023 (PMC8392180; doi:10.3390/biomedicines9081023)
Supplement: Supplementary file 1 [file biomedicines-09-01023-s001.zip › biomedicines-1313677-supplementary.pdf]

Supporting Information

Plasma-based Bioinks for Extrusion Bioprinting of Advanced Dressings

Cristina Del Amo, Arantza Perez-Valle, Miguel Perez-Garrastachu, Ines Jauregui, Noelia Andollo, Jon Arluzea, Pedro Guerrero, Koro De la Caba and Isabel Andia[*]

Initial printability screening was performed using filament drop tests and rheological evaluations for different modifications of blends (data not shown). To determine the optimal extrudability parameters, we used a representative complex pattern of the internal structure of the final constructs (Figure S1). Overall, 24 printing configurations were systematically tested, including variations of the extrusion multiplier (1.0 and 1.1), speed (1.0, 1.5, 2.0, 2.5, and 3.0 mm/s), and layer height (0.2, 0.3, 0.4, 0.5, and 0.6 mm), using PRP-based bioinks. Print geometries were examined using a binocular Nikon magnifier (C-DSD230), and images were analyzed using ImageJ/Fiji software (National Institutes of Health, Bethesda, MD, USA). To assess the extrusion measurement, we compared experimental bioprints with the theoretical geometry (40 mm^2) (Equation S1).

$$Extrusion\ measurement\ = \left|\frac{Theoretical\ dimension - Measured\ dimension}{Theoretical\ dimension}\right| \times 100 \quad \text{(S1)}$$

The closer the extrusion index is to zero, the closer the shape is to the theoretical dimensions of the geometry.

Table S1. Systematic variation in the bioprinter parameters used to establish the manufacturing process

**Formulation**	**Material flow (%)**	**Height (mm)**	**Speed (mm·s^{-1})**	**Extrusion measurement**
PRP/ALG	**100**	0.2	1	25.47
			2	15.75
			3	6.63
		0.3	1	56.44
			2	18.63
			3	17.42
		**0.4**	1	38.05

			1.5	42.26
			2	12.40
			2.5	5.09
			**3**	**3.25**
		0.5	1	9.69
			2	40.27
			3	30.24
		0.6	1	18.78
			2	30.14
			3	19.47
	110	0.4	1	33.18
			2	27.25
			3	16.95
Pristine ALG	100	0.4	3	26.39
Ultrasound gel	100	0.4	3	12.06
NIVEA	100	0.4	3	2.39
PLA	100	0.4	3	0.73

The Nivea crème and ultrasound gel (Transonic; Promofarma) were used as demonstration inks.[16,47] Additionally, we used polylactic acid (Smartfil PLA; Smartmaterials3d, Jaén, Spain), which is a common polymeric material in extrusion-based 3D printing, as a control to compare the levels of structural differences using a common 3D printer (0.6-mm nozzle Ultimaker 2+; Ultimaker, Utretch, Netherlands).

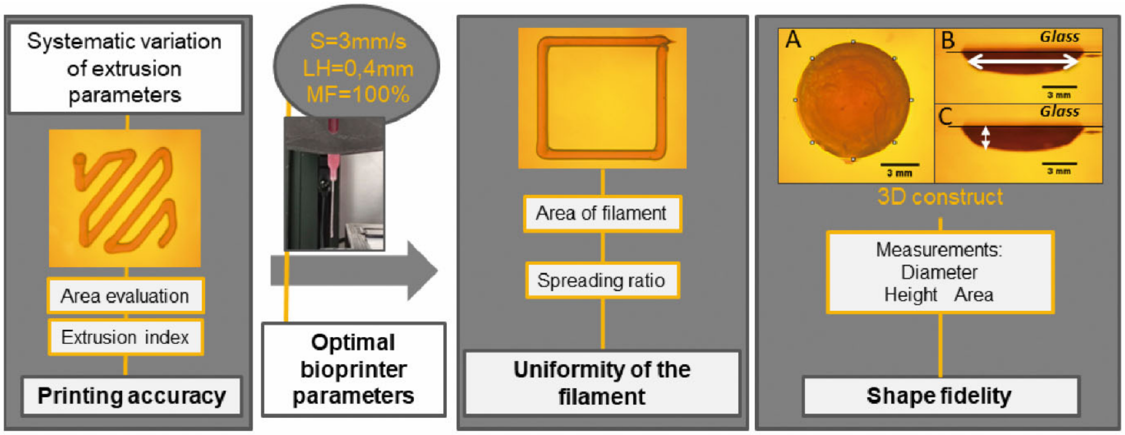

Figure S1. Flowchart of the experiments performed to analyze printing outcomes. (1) Systematic variation of extrusion parameters to set up the bioprinting process with the PRP bioink. The layout was defined as a single layer of a squared geometry at a 10-mm width with a 40% infill without perimeters using free software for computer-aided design (TinkerCAD; AutoDesk, Mill Valley, CA, USA). The exported 0.stl file was uploaded into Simplify3D software (3D printer slicer; Simplify3D, Cincinnati, OH, USA) to define bioprinting parameters. Once all the variables were defined, the .G-code file with the optimal printing instructions was exported and executed in the bioprinter. (2) Analyses of extruded filaments with PPP- and PRP-based bioinks. (3) Analyses of the shape fidelity of 3D scaffolds (structural differences between the design and hydrogel prints): (A) area of the base, (B) cross-sectional diameter, and (C) mean height. Bioprinting outcomes were compared between PRP and PPP bioinks. LH: layer height; MF: material flow; S: speed.

Shape fidelity

To evaluate the uniformity of the filament, a simple pattern was substituted for the complex geometry (Figure S1.2). The filament shapes obtained with PRP- and PPP-based plasma inks were 54.04 ± 6.23 versus 45.01±5.64 mm^2, respectively ($p<0.001$), which differed from the experimental measurements obtained for the filaments of the demonstration inks (21.77 mm^2 for Nivea®, 32.33 mm^2 for PLA, 31.54 mm^2 for the ultrasound gel, and 38.28 mm^2 for alginate, autoclaved as a solution). Except for Nivea®, the demonstration inks also showed divergence from the ideal printing outcome (the theoretical area of the filament was 22.56 mm^2).

The three-dimensional fidelity (structural differences between the theoretical 3D design and bioprints) for multilayered constructs was studied by printing circular disks (10 mm width; 1.6 mm height; theoretical area: 78.57 mm^2; and theoretical volume: 125.7 mm^3). The real dimensions of the constructs are shown in Figure S1.3. The experimental areas of the PRP and PPP prints were 80.21 ± 3.81 and 80.75 ± 6.78 mm^2, respectively (Figure S1.3A). There were no significant differences between the PRP and PPP prints in the diameter measured in the middle section (9.15 ± 0.40 and 9.45 ± 0.31 mm, respectively) (Figure S1.3B). The experimental heights for the PRP and PPP prints were 1.77 + 0.16 and 1.80 + 0.14 mm, respectively, with no significant difference (Figure S1.3C). The filaments were merged with both bioinks, resulting in a fully filled construct.
